# Noninvasive Positive Pressure Ventilation (NIPPV)-Associated Expanding Hiatal Hernia Causing Pulmonary Tamponade: A Case Report on Unusual Complication of NIPPV

**DOI:** 10.7759/cureus.25069

**Published:** 2022-05-17

**Authors:** Ashish Jain, Maha Mumtaz, Khandakar M Hussain, Asfandyar Butt, Zonghao Pan

**Affiliations:** 1 Internal Medicine, Conemaugh Memorial Medical Center, Johnstown, USA

**Keywords:** continuous positive airway pressure (cpap), cpap, hypoxic respiratory failure, atelectasis, s:hiatal hernia, noninvasive positive pressure ventilation

## Abstract

Numerous diseases related to gastric distension have been found and shown to be linked with noninvasive positive pressure ventilation (NIPPV). We describe the case of a 93-year-old female who came with progressively worsening shortness of breath that initially responded to NIPPV but subsequently deteriorated. Imaging revealed gaseous distension of a preexisting hiatal hernia with air-fluid levels and compressive effects on the left lower lobe of the lung. She was successfully managed using a conservative decompression strategy. This is the first case to our knowledge of NIPPV causing considerable distension of an existing hiatal hernia to the point of mediastinal tamponade.

## Introduction

Historically, non-invasive ventilation has been used to treat respiratory insufficiency in patients with heart failure and chronic obstructive and restrictive pulmonary diseases to reverse pulmonary edema and improve respiratory status. The positive intrathoracic pressure provided by non-invasive positive pressure ventilation (NIPPV) has an effect on the patient's hemodynamic status by reducing cardiac preload and afterload by decreasing venous return to the heart. Gastric and intestinal distension, and in rare cases perforation, is a well-documented complication of NIPPV. They are thought to occur due to continuous positive airway pressure (CPAP) serving as a channel for continuous air entry into the stomach. Our case demonstrates that hiatal hernia may result in gaseous distension of the intrathoracic stomach and can manifest as an acute deterioration of respiratory status in such cases. Although nasogastric decompression is a reasonable mode of management in such circumstances, a conservative decompression trial should be performed first by withdrawing the NIPPV support.

## Case presentation

**Figure 1:**
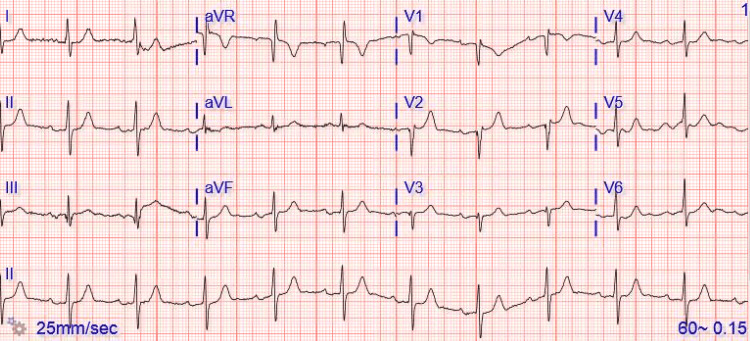
EKG demonstrating sinus rhythm with first-degree atrioventricular block.

We present the case of a 93-year-old female with a history of heart failure with preserved ejection fraction (HFpEF), bioprosthetic aortic and mitral valve replacement, recurrent pneumonia, and chronic obstructive pulmonary disease on home oxygen with progressively worsening shortness of breath and fever for three days. She was put on CPAP, which initially aided the patient in breathing. Initial vital signs revealed a blood pressure of 139/59 mm/hg, heart rate of 70 beats per minute, a temperature of 104 degrees Fahrenheit, and respiratory rate of 24 beats per minute. On presentation, an electrocardiogram revealed sinus rhythm with first-degree atrioventricular block and signs of inadequate R-wave progression across the mid-precordial leads, suggestive of an old anteroseptal infarct (Figure 1).

**Figure 2:**
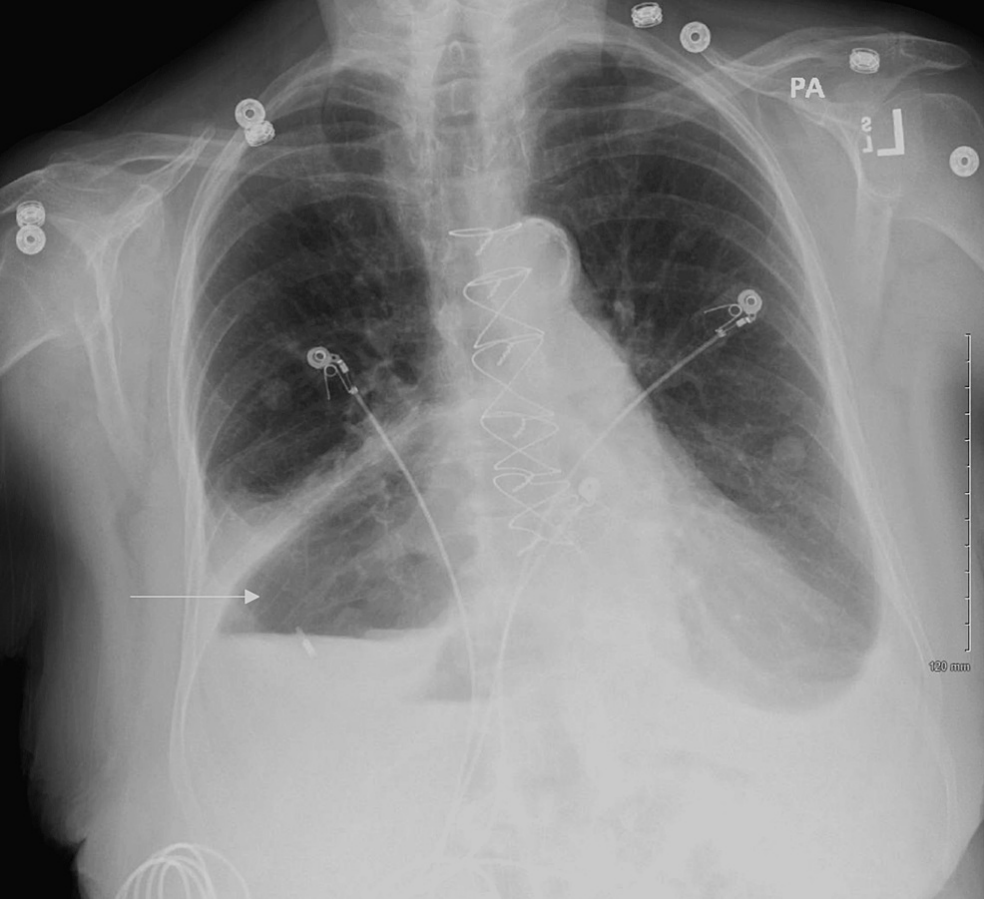
Chest X-ray in the PA view showing a large hiatal hernia with gaseous distention of the intrathoracic stomach. PA: Posterior anterior.

Initial laboratory results revealed a lactic acid level of 2.2 mmol/L, a creatinine level of 1.6 mg/dL, a B-type natriuretic peptide (BNP) level of 371 pg/mL, and leukocytosis of 9.7 X 10*3/uL. Although the patient met systemic inflammatory response syndrome (SIRS) criteria for sepsis, a liberal fluid resuscitation regimen was not implemented because of apparent fluid overload and the suspicion of an acute exacerbation of congestive heart failure. However, she received the first dosage of vancomycin and piperacillin-tazobactam. Following an initial response to CPAP, the patient's respiratory condition began to deteriorate. She was sent for a chest X-ray which showed a large hiatal hernia with gaseous distention of the intrathoracic stomach (Figure 2).

**Figure 3:**
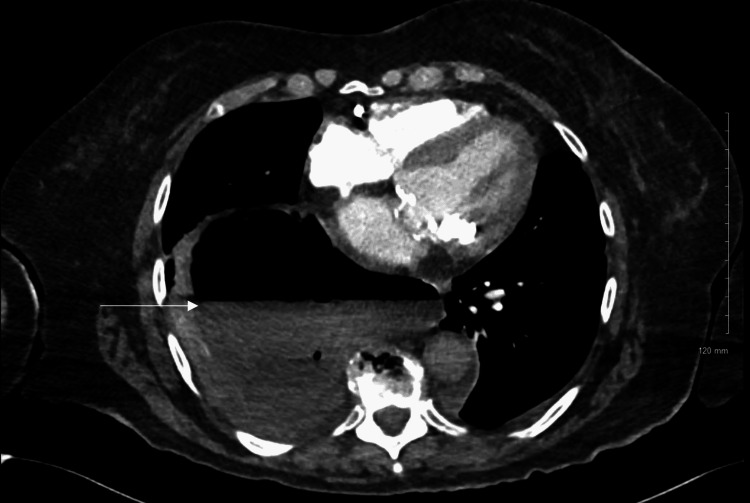
CT angiogram of the chest demonstrating large hiatal hernia with air-fluid levels in the axial plane.

**Figure 4:**
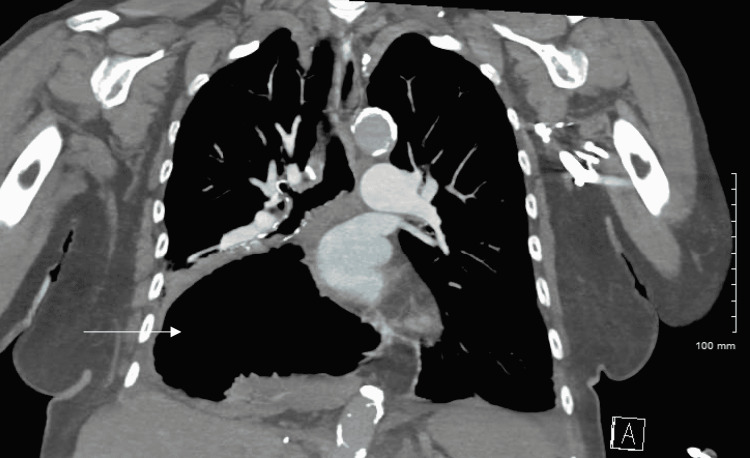
CT angiogram of the chest demonstrating large hiatal hernia with air-fluid level in the coronal plane.

However, a CT angiogram of the chest, which ruled out pulmonary embolism, revealed a massive hiatal hernia compressing the right lower and middle lobes (Figures 3-4). Positive end-expiratory pressure with CPAP was thought to cause aerophagia, or air eating, resulting in the ballooning of the hiatal hernia in the thorax.

**Figure 5:**
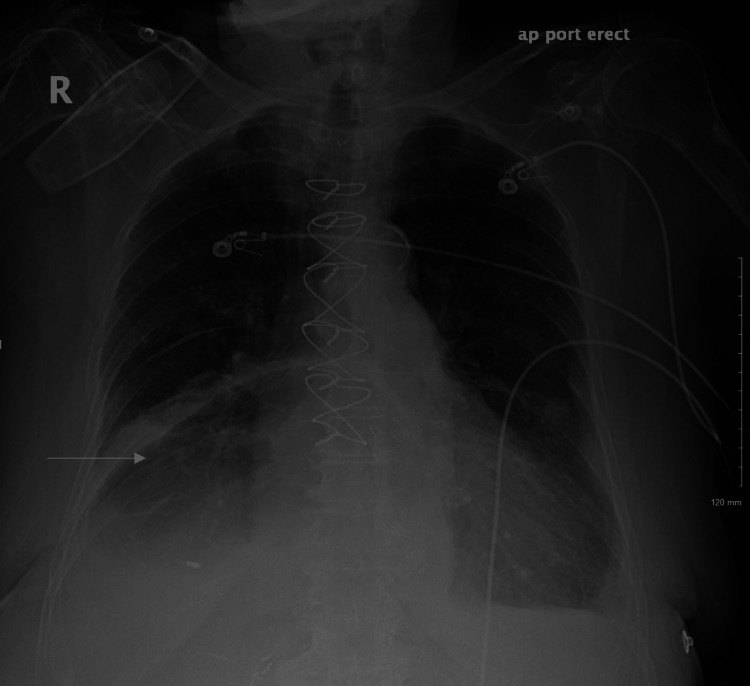
Chest X-ray in the anteroposterior view demonstrating right lower lobar consolidation and right-sided hiatal hernia.

A conservation gastric decompression strategy was adopted, and the patient was weaned off CPAP and switched to a high-flow nasal cannula. The patient's respiratory status improved steadily, and a repeat chest X-ray revealed evidence of a hiatal hernia, although it had substantially decreased in size (Figure 5).

Her blood cultures demonstrated Streptococcus mitis, for which she was transitioned to ceftriaxone. Given her bioprosthetic mitral and aortic valve replacement history, she had transesophageal echocardiography, which revealed no indication of vegetations on either mitral or aortic valve. She was eventually discharged from the hospital with a peripherally inserted central line for six weeks of outpatient ceftriaxone therapy.

## Discussion

Hiatus hernia is a term that refers to disorders in which components of the abdominal cavity, most often the stomach, herniate into the mediastinum via the esophageal hiatus. Only the esophageal hiatus is susceptible to visceral herniation of the diaphragm apertures because it opens directly into the abdominal cavity and is therefore immediately exposed to the pressure strains between the two cavities. Notably, the esophagus does not completely cover the gap due to the need for expansion to accommodate luminal contents [1]. Due to the anatomy of the esophagogastric junction and a weak lower esophageal sphincter tone as seen in our patient, with a chronic history of gastroesophageal reflux disease and continuous post-end expiratory pressure may gradually raise intragastric pressure by serving as a channel for continuous air entry in the stomach. Air swallowing or aerophagia may occur as a psychogenic phenomenon due to aberrant anatomy or as a side effect of NIPPV. Excessive air in the stomach may have a detrimental effect on the respiratory and GI systems. Aerophagia, which is sometimes confused with air swallowing, is a distinct behavioral disorder. Regardless of the mechanism through which air enters the stomach, it has been observed that an unmitigated buildup of intraluminal gas might impair breathing or result in mediastinal tamponade in a way similar to tension pneumothorax [2]. Numerous pathologies associated with gastric distension have been identified and reported to be associated with NIPPV, but to our knowledge, this is the first case in which NIPPV resulted in significant distension of an existing hiatal hernia to the point of mediastinal tamponade. Although Adrian Regli's research demonstrated that non-invasive ventilation was not correlated with an increase in intra-abdominal pressure during a 72-hour period [3], cases of postoperative air leakage during NIPPV support have been made. Jean-Lavaleur M et al. described a case of gastric perforation with NIPPV; the patient had a full recovery after primary closure [4]. Vasquez TL and Hoddinott K describe two patients treated with BIPAP postoperatively after open Roux-en-Y gastric bypass (RYGBP) and had considerable bowel distention and subsequent anastomotic leakage [5]. Although nasogastric decompression was an acceptable method of gastric decompression in our case, we initially chose a conservative strategy for our patient due to the high risk of gastric rupture resulting in frank mediastinitis and the nasogastric tube placement being an extremely uncomfortable procedure for the patient. If possible, we advocate for a tiered approach to intervention in such situations. It is preferable to begin with a minimally invasive strategy and move to a more invasive intervention only if the less invasive strategy fails.

## Conclusions

In conclusion, sudden deterioration of respiratory status in patients receiving non-invasive positive pressure ventilation, or NIPPV, has long been associated with pulmonary barotrauma and pneumothorax. Albeit uncommon, intrathoracic distension of a preexisting hiatal hernia might appear similarly. Although nasogastric decompression is an appropriate therapeutic strategy, if possible, we recommend a graded approach in such patients, beginning with the removal of NIPPV support.
